# Longitudinal adherence to maternal antiretroviral therapy and infant Nevirapine prophylaxis from 6 weeks to 18 months postpartum amongst a cohort of mothers and infants in South Africa

**DOI:** 10.1186/s12879-019-4341-4

**Published:** 2019-09-16

**Authors:** Anna Larsen, Vuyolwethu Magasana, Thu-Ha Dinh, Nobubelo Ngandu, Carl Lombard, Mireille Cheyip, Kassahun Ayalew, Witness Chirinda, Gurpreet Kindra, Debra Jackson, Ameena Goga

**Affiliations:** 1 Division of Global HIV/AIDS and Tuberculosis, Center for Global Health, US Centers for Disease Control and Prevention (CDC), Pretoria, South Africa; 2 Health Systems Research Unit, South African Medical Research Council (HSRU, SAMRC), Pretoria, South Africa; 3 Division of Global HIV/AIDS and Tuberculosis, Center for Global Health, US Centers for Disease Control and Prevention (CDC), Atlanta, GA USA; 4 United Nations Children’s Fund (UNICEF), New York, NY USA; 5 School of Public Health, University of the Western Cape, Cape Town, South Africa; 6 Department of Paediatrics, University of Pretoria, Pretoria, South Africa; 7 HIV Prevention Research Unit, South African Medical Research Council, 123 Jan Hofmeyr Road, Westville, Durban, KwaZulu-Natal 3630 South Africa

**Keywords:** HIV-exposed infants, Postnatal care, Missed visits

## Abstract

**Background:**

Despite improved policies to prevent mother-to-child HIV transmission (MTCT), adherence to maternal antiretroviral therapy (ART) and infant Nevirapine prophylaxis (NVP) is low in South Africa. We describe ART adherence amongst a cohort of HIV-positive mothers and HIV-exposed but uninfected infants from 6 weeks until 18 months post-delivery and identify risk factors for nonadherence.

**Methods:**

Data were collected in 2012–2014 through a nationally representative survey of PMTCT effectiveness. Mother-infant pairs were enrolled during the infant’s first immunization visit at 6 weeks. Mothers and HIV-exposed infants (2811 pairs) were followed to 18 months at 3-month intervals. Mothers who self-reported being on ART at 6 weeks postpartum (*N* = 1572 (55.9%)) and infants on NVP at 6 weeks (*N* = 2370 (84.3%)) were eligible for this analysis and information about their adherence was captured at each interview they attended thereafter. We defined nonadherence within each 3-month interval as self-report of missing > 5% of daily ART/NVP doses, estimated adherence using a Cox survival curve with Andersen & Gill setup for recurring events, and identified risk factors for nonadherence with an extended Cox regression model (separately for mothers and infants) in Stata 13. Results are not nationally representative as this is a subgroup analysis of the follow-up cohort.

**Results:**

Amongst mothers on ART at 6 weeks postpartum, cumulative adherence to maternal ART until 18 months was 63.4%. Among infants on NPV at 6 weeks postpartum, adherence to NVP was 74.5%.. Risk factors for nonadherence to maternal ART, controlling for other factors, included mother’s age (16–24 years vs. ≥34 years, adjusted Hazard Ratio (aHR): 1.9, 95% CI: 1.4–2.5), nondisclosure of HIV status to anyone (nondisclosure vs. disclosure: aHR: 1.7, 95% CI: 1.3–2.1), and timing of ART initiation (initiated ART after delivery vs. initiated ART before delivery: aHR: 1.6, 95% CI: 1.3–2.0). Provincial variation was seen in nonadherence to infant NVP, controlling for other factors.

**Conclusion:**

Maintaining ART adherence until 18 months postpartum remains a crucial challenge, with maternal ART adherence among the six week maternal ART cohort below 65% and infant NVP adherence among breastfeeding infants in this cohort below 75%.This is gravely concerning, given the global policy shift to lifelong ART amongst pregnant and lactating women, and the need for extended infant prophylaxis amongst mothers who are not virally suppressed. Our findings suggest that young mothers and mothers who do not disclose their status should be targeted with messages to improve adherence, and that late maternal ART initiation (after delivery) increases the risk of maternal nonadherence.

## Introduction

Since the introduction of triple antiretroviral treatment (ART) and extended infant prophyaxis by national-level programs to prevent mother to child transmission of HIV (PMTCT) each new generation of infants in Sub-Saharan Africa has been born with lower prevalence of HIV than the last [1, 2]. Highly active antiretroviral treatment (ART) preserves a mother’s overall health, reduces her viral load, and reduces risk of MTCT, while Nevirapine (NVP) as infant HIV prophylaxis prevents infection in HIV-exposed infants [3, 4]. Global gains in PMTCT have reduced transmission such that numerous countries imminently face elimination of maternal to child transmission of HIV (eMTCT)- achieving generations born HIV-free [5, 6].

Despite these gains, an estimated 122,000 children under the age of 15 years acquired HIV infections in sub-Saharan Africa in 2015, most of which were due to MTCT [7]. South Africa remains the country with the highest number of people living with HIV (PLHIV) in the world, 6.4 million PLHIV [8], accounting for 18% of global HIV prevalence [9]. HIV prevalence among women reporting to public health facilities for antenatal care (ANC) is high at 29.7% in 2013 [10], demonstrating the magnitude of the PMTCT program required to maintain low MTCT in South Africa.

South Africa has aggressively adopted the World Health Organization’s (WHO) increasingly rigorous guidelines for PMTCT since introducing a national PMTCT program in 2002 [11, 12]. Impressively, South Africa reduced early MTCT at 4–8 weeks postpartum from 9% in 2009 to 2.6% (95% CI 2.0–3.2) in 2012–2013 [13].

Under WHO Option A and Option B (adopted in April 2013), all HIV-positive pregnant and breastfeeding women were entitled to ART for life if eligible by a CD4 count less than 350 cells/mm^3^. Under Option A, pregnant women with a CD4 count over 350 cells/mm^3^ were eligible for ART prophylaxis from 14 weeks gestation until 7 days postpartum. HIV-exposed infants received daily NVP from birth to 7 days after cessation of breastfeeding or through age 4–6 weeks if the mother was not breastfeeding. Under Option B, pregnant women with a CD4 count over 350 cells/mm^3^ were eligible for triple ART from 14 weeks gestation through 7 days after cessation of breastfeeding and all HIV-exposed infants were provided daily NVP through age 4–6 weeks regardless of feeding method [14, 15]. In December 2014, South Africa adopted Option B+ entitling all HIV positive pregnant and lactating women ART for life regardless of clinical indicators [16]. With these efforts, more than 91% of HIV-positive mothers in South Africa received ART or prophylaxis in 2014 [17].

Despite high coverage of the PMTCT program and successful reduction in MTCT, the first HIV-free generation in South Africa will only be possible once factors influencing ongoing MTCT are alleviated. One such factor - nonadherence to ART among mothers and infants from pregnancy to cessation of breastfeeding - poses a particular threat to MTCT rates by reducing viral suppression and enabling transmission [18]. By understanding the magnitude of nonadherence to ART among mothers and infants and the risk factors for nonadherence, we can inform programmatic shifts to reduce nonadherence and MTCT.

Nonadherence is difficult to measure as it often relies on information that is self-reported by a patient. Concerns remain about the validity of self-reported adherence measures due to memory biases which often overestimate adherence [19]. However, several studies substantiate these measures as appropriate for estimating adherence to ART in developing settings [20, 21]. While there is no gold standard metric [22], ART adherence between 80 and 99% is critical to maintain viral suppression, reduce risk of developing drug resistance, and reduce risk of MTCT [23]. Much of the literature regarding ART adherence, sets the threshold for adherence as achieving at least 95% of prescribed daily doses since this is required for optimal health outcomes [24]. The analyses herein utilize self-reported information to assess 95% adherence to ART among mothers and infants.

We describe longitudinal, self-reported adherence to ART among HIV-positive mothers and to Nevirapine among their HIV-exposed infants at 3-month intervals from 6 weeks to 18 months postpartum within a subgroup of the follow-up cohort for PMTCT evaluation. Further, we identify risk factors for nonadherence with the goal of informing improvements to PMTCT programming that support ART adherence among mother-infant pairs.

## Methods

### Study design and population

This study was a secondary analysis of data collected to evaluate the effectiveness of the national programme to prevent HIV transmission from mother to child in South Africa. The evaluation was a nationally-representative cross-sectional survey conducted in 2012/13 (October 2012–May 2013), with infant follow-up until September 2014. Public sector health facilities were sampled using multi-stage, probability proportional to size methodology using three strata per province based on 6 week client immunization load and antenatal HIV prevalence. The study was powered to produce nationally-representative results of MTCT at 6 weeks. More detailed information about the survey is available in previous publications [11, 13].

Mother-infant pairs were enrolled into the study during the infant’s first postpartum immunization visit at 6 weeks as national coverage for this first postpartum immunization visit is known to be over 60% in surveyed areas of South Africa [25]. If the infant was deemed HIV exposed but uninfected at the 6 week cross-sectional visit, mother-infant pairs [*N* = 2811] were eligible and consented for a further cohort survey involving three-monthly follow-up, from 3 until 18 months. The primary outcomes of this 3-to-18 month national cohort will be presented in a separate publication.

### Data and blood collection

Trained nurse fieldworkers interviewed mothers or non-mother caregivers at the 6 week immunization visit and follow-up visits conducted at 14 weeks and six, nine, twelve, fifteen, and 18 months postpartum about socio-demographic information, infant health and feeding practices, maternal understanding of MTCT, HIV testing, postnatal care, and maternal and infant PMTCT prophylaxis and treatment. Approximately 375 μl of blood per infant was drawn and dropped onto five pre-printed circles on Munktell TFN filter paper to determine infants HIV exposure and infection. Visual aids such as pictures of ARV pills and ARV syrup bottles were used to assist mothers in identifying the type of regimen received. A feeding and medication diary was issued to mothers so they could document medication received, and to assist with recall of missed daily doses during interviews. Non-mother caregivers were not asked questions about maternal health and/or HIV testing and treatment.

### Data analysis

Two analyses were conducted: Maternal ART adherence (ART analysis) amongst mothers eligible for analysis and infant nevirapine (NVP) adherence (NVP analysis) amongst HIV-exposed infants eligible for analysis. Mothers were eligible for inclusion in the ART analysis if they were included in the follow-up cohort of 2811 mother-infant pairs and indicated that they were on ART at 6 weeks post-delivery (*N* = 1572). At each follow-up interval thereafter at 14 weeks, 6, 9, 12, 15, and 18 months postpartum, information about ART adherence was included in the analysis for mothers (not other caregivers) who attended that particular interview and were still taking ART.

Infants were eligible for inclusion in the NVP analysis if they were HIV-exposed, included in the follow-up cohort of 2811 mother-infant pairs and were reported to be on infant NVP at the six week visit, (*N* = 2370). Thereafter, information about NVP adherence was included in the analysis for breastfeeding infants who attended that particular interview and were still on NVP. Mothers and infants who were removed from ART or NVP based on national guidelines were not further analyzed for adherence in this analysis.

Nonadherence to ART among mothers and NVP among infants was defined as reporting taking less than 95% of daily doses within each three month follow-up interval. For mothers and infants, this was operationalized in two scenarios where some mothers/infants were deemed non-adherent because they had failed to take ART/NVP for more than 5% of the daily doses in that interval, while others were non-adherent because they had ceased taking ART/NVP within the interval without professional advice. We assumed that medical advice to stop NVP was provided in compliance with national guidelines (therefore indicating adherence). We assumed other reasons for stopping NVP were not in compliance guidelines (therefore indicating nonadherence).

For mothers or infants who reported missing more than 5% of daily doses, failure date was calculated as the midpoint of the interview interval. For infants who stopped taking NVP during an interval, their age in weeks at NVP cessation was used to calculate date of failure.

Mothers were excluded from the adherence analysis at a particular time point if they had missed an interview during the interval of interest or a non-mother caregiver had responded to the interview, thus information about the mother’s adherence was not provided. Infants were no longer included in the analysis if breastfeeding had been stopped in the prior interval, thus removing the threat of transmission and the need for NVP per PMTCT guidelines.

We performed frequency analyses to describe characteristics of the mothers and infants and their care. To describe probability of ART or NVP adherence from 6 weeks to 18 months postpartum we plotted extended Cox model survival curves using the stcox and stcurve commands in Stata [26, 27] after preparing the data for Andersen-Gill time-to-event analysis for recurring events [28, 29]. The Andersen-Gill model is a counting process allowing assessment of multiple nonadherence events across follow-up intervals instead of censoring on the first instance of nonadherence [28, 30, 31].

We fitted multivariable Andersen-Gill extended Cox models to determine risk factors for ART nonadherence among mothers and infants separately. Andersen-Gill models generalize the Cox proportional hazards model, accommodating the non-proportionality of the effect of covariates in the case of recurrent failure events [31]. The resulting coefficients are a measure of all-cause effects across multiple recurrent events as demonstrated in other studies describing risk factors associated with recurring health events [32–35].

Covariates of interest from the information collected at 6 weeks were identified a priori. We used the forward stepwise function to fit the models from these covariates, setting the significance level for addition to the model at 15% and removal from the model at 20% [30]. The proportional hazards assumption was upheld for both models which we assessed by running a model that included time-varying covariates. All analyses were performed in STATA 13.1. Because this is a subgroup analysis of the cohort of mothers followed up by the study, the results are not nationally representative. Therefore, results are not adjusted for the study design, non-response and not weighted for live-births.

### Ethical consideration

The protocol was reviewed according to the Centers for Disease Control and Prevention (CDC) human research protection procedures and was determined to be research, but CDC was not engaged. The protocol was also reviewed by the institutional review board of the Medical Research Council of South Africa. Mothers and caregivers provided written informed consent prior to the onset of the interview and assent for infants to undergo blood collection.

## Results

### Characteristics of study population

The majority of the mothers in the study population were between 25 and 34 years old (ART analysis: 59.2%, NVP analysis: 56.3%). Three quarters of the mothers in both samples were educated up to grade 8–12. Most mothers were single (ART analysis: 70.8%, NVP analysis: 73.9%) (Table 1).

**Table 1:** Characteristics of mothers (*N* = 1572) on ART at 6 weeks postpartum and infants (*N* = 2370) on NVP at 6 weeks postpartum among a cohort in South Africa in 2012–2014

Characteristics	Mothers on ART at 6 weeks *N* = 1572	Infants on NVP at 6 weeks *N* = 2370
	N (%)	N (%)
MATERNAL CHARACTERISTICS		
Maternal age		
16–24 years	298 (19.0)	619 (26.1)
25–34 years	931 (59.2)	1334 (56.3)
35+ years	343 (21.8)	417 (17.6)
Education of mother		
Grades 1–7	297 (18.9)	410 (17.3)
Grades 8–12	1196 (76.1)	1827 (77.1)
Tertiary	53 (3.4)	94 (4.0)
None/DK/NA	26 (1.6)	39 (1.6)
Marital status		
Single	1113 (70.8)	1751 (73.9)
Married	295 (18.8)	395 (16.7)
Co-habiting	151 (9.6)	207 (8.7)
Widowed	10 (0.6)	13 (0.6)
Divorced/separated	3 (0.2)	4 (0.2)
Province		
Northern Cape	60 (3.8)	76 (3.2)
Limpopo	148 (9.4)	233 (9.8)
Eastern Cape	143 (9.1)	206 (8.7)
Free State	222 (14.1)	301 (12.7)
Gauteng	312 (19.9)	471 (19.9)
KwaZulu Natal	252 (16.0)	407 (17.2)
Mpumalanga	151 (9.6)	266 (11.2)
North West	139 (8.9)	201 (8.5)
Western Cape	145 (9.2)	209 (8.8)
Primary source of income		
Other^a^	1225 (77.9)	1910 (80.6)
Own employment	347 (22.1)	460 (19.4)
Parity		
One child	323 (20.6)	562 (23.7)
2–3 children	983 (62.5)	1453 (61.3)
4+ children	266 (16.9)	355 (15.0)
Transport to clinic		
Walk	892 (56.7)	1309 (55.2)
Taxi/Bus/Train	603 (38.4)	956 (40.3)
Own vehicle	68 (4.3)	89 (3.8)
Other	9 (0.6)	16 (0.7)
Time to reach clinic (minutes)		
0–30 min	1280 (81.4)	1919 (81.0)
> 30 min	292 (18.6)	451 (19.0)
PREGNANCY AND CONTINUUM OF CARE		
Planned pregnancy		
No	936 (59.5)	1491 (62.9)
Yes	633 (40.3)	879 (37.1)
DK/NA	3 (0.2)	0 (0.0)
Timeliness of first ANC visit		
> 12 weeks	1030 (65.5)	1589 (67.1)
≤ 12 weeks	542 (34.5)	781 (32.9)
ANC Visits		
< 4 visits	156 (9.9)	286 (12.1)
4+ visits	1416 (90.1)	2084 (87.9)
Delivery care location		
Hospital	1246 (79.3)	1830 (77.2)
Clinic	283 (18.0)	456 (19.3)
Home/other	43 (2.7)	84 (3.5)
PNC Visits		
< 3 visits	1269 (80.7)	1885 (79.5)
3+ visits	303 (19.3)	485 (20.5)
Ever breastfed infant in first 6 weeks		
No	1005 (63.9)	1485 (62.7)
Yes	567 (36.1)	885 (37.3)
HIV TESTING AND TREATMENT FACTORS		
HIV status disclosed to family and/or friends		
No	177 (11.3)	400 (16.9)
Yes	1395 (88.7)	1970 (83.1)
Aware of CD4 cell count		
CD4 test done and result received	1037 (65.9)	1519 (64.1)
CD4 test not done	49 (3.1)	119 (5.0)
CD4 test done, result not received	441 (28.1)	668 (28.2)
Do not know if had CD4 test	45 (2.9)	64 (2.7)
Ever heard of PMTCT		
No	192 (12.2)	288 (12.2)
Yes	1380 (87.8)	2082 (87.8)
Initiated ART after delivery		
No	599 (38.1)	691 (29.1)
Yes	783 (49.8)	1457 (61.5)
Don’t know/Chose not to answer	190 (12.1)	222 (9.4)

^a^ Other primary sources of income include: Child support grant, disability grant, partner/husband/ex-husband, other family member

At the six week interview, most mothers reported a main source of income other than their own employment such as a child support grant, disability grant, a partner or husband’s support, or another family member’s support (ART analysis: 77.9%, NVP analysis: 80.6%). Over a third of infants (37.3%) were breastfed between birth and 6 weeks. Less than half of the mothers had planned pregnancies (ART analysis: 40.3%, NVP analysis: 37.1%).

### Adherence to antiretroviral treatment among mothers and infants

**Fig. 1:**
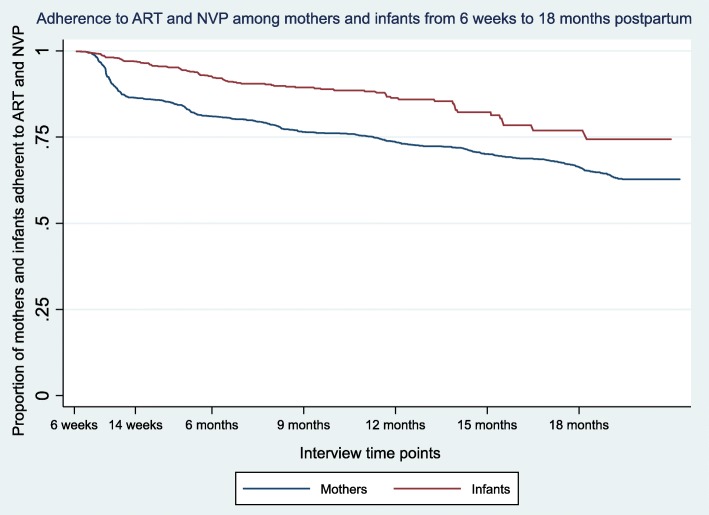
Adherence to ART among a cohort of mothers (*N* = 1572) and NVP among a cohort of infants (*N* = 2370) at 6 weeks, in South Africa and followed-up at 3-month intervals from 6 weeks to 18 months postpartum in 2012–2014 (unweighted)

Cumulative probability of adherence to ART until 18 months postpartum was 63.4% among mothers included in the ART analysis (95% confidence interval (CI): 60.7–66.0) and 74.5% among infants included in the NVP analysis (95% CI: 70.2–81.9) (Fig. 1, Table 2). From 6 weeks to 14 weeks postpartum, adherence to NVP was 96.4% among infants (95% CI: 94.3–97.8). From 6 weeks to 14 weeks postpartum, ART adherence was lower amongst mothers at 85.0% (95% CI: 82.8–87.0). Cumulative infant NVP adherence was consistently higher than mother ART adherence over the 6 weeks to 18 months postpartum period.

**Table 2:** Frequency of nonadherence types per interval and cumulative probability of adherence to ART among a cohort of mothers (*N* = 1572) and NVP among a cohort of infants (*N* = 2370) from 6 weeks to 18 months postpartum in South Africa followed in 2012–2014 (unweighted)

	Follow-up intervals from 6 weeks to 18 months postpartum					
	6 weeks - 14 weeks	14 weeks - 6 months	6 months - 9 months	9 months - 12 months	12 months - 15 months	15 months - 18 months
Adherence to ART among mothers reported on ART						
Total number of mothers entering interval	*n* = 1026	*n* = 902	*n* = 866	*n* = 833	*n* = 800	*n* = 832
Cumulative probability of adherence^a^	85.0 (82.8–87.0)	79.1 (76.6–81.4)	74.2 (71.6–76.6)	71.1 (67.0–72.2)	67.3 (64.6–69.9)	63.4 (60.7–66.0)
Total adherence events during interval	861	820	801	777	749	763
Total nonadherence events during interval	165	82	65	56	51	69
Taking ART but missed > 5% doses	25 (15.2)	17 (20.7)	17 (26.2)	9 (16.1)	8 (15.7)	8 (11.6)
Stopped taking ART during interval (not according to care)	140 (84.8)	65 (79.3)	48 (73.8)	47 (83.9)	43 (84.3)	61 (88.4)
Mothers not included in the analysis at each stage and reason for exclusion						
Total number of mothers not reporting on ART at interval	*n* = 546	*n* = 670	*n* = 706	*n* = 739	*n* = 772	*n* = 740
Missed interview	462 (84.6)	508 (75.8)	528 (74.8)	544 (73.6)	542 (70.2)	473 (63.9)
Non-mother caregiver gave interview	84 (15.4)	92 (13.7)	94 (13.3)	111 (15.0)	150 (19.4)	185 (25.0)
No longer on ART	0 (0.00)	70 (10.5)	84 (11.9)	84 (11.4)	80 (10.4)	82 (11.1)
Adherence to NVP among infants reported on NVP						
Total number of infants assessed for adherence in interval	*n* = 1145	*n* = 702	*n* = 426	*n* = 248	*n* = 125	*n* = 52
Cumulative probability of adherence^a^	96.4 (94.3–97.8)	92.3 (89.8–94.2)	88.8 (85.9–91.2)	86.9 (83.6–89.6)	81.6 (76.9–85.5)	74.5 (70.2–81.9)
Total adherence events during interval	1102	670	403	236	113	49
Total nonadherence events during interval	43	32	23	12	12	3
Taking ART but missed > 5% doses	8 (18.6)	12 (37.5)	5 (21.7)	3 (25.0)	5 (41.7)	0 (0.0)
Stopped taking NVP during interval (not according to care)	35 (81.4)	20 (62.5)	18 (78.3)	9 (75.0)	7 (58.3)	3 (100.0)
Infants not included in the analysis during each interval and reason for exclusion						
Total number of infants not reporting on NVP at interval	*n* = 1225	*n* = 1668	*n* = 1944	*n* = 2122	*n* = 2245	*n* = 2318
Missed interview	338 (27.6)	405 (24.3)	437 (22.5)	448 (21.1)	425 (18.9)	389 (16.8)
Appropriately stopped NVP	887 (72.4)	1263 (75.7)	1507 (77.5)	1674 (78.9)	1821 (81.1)	1929 (83.2)
Never breastfed during interval	850 (95.8)	1183 (93.7)	1404 (93.2)	1542 (92.1)	1678 (92.2)	1784 (92.5)
Clinician said to stop NVP	37 (4.2)	80 (6.3)	103 (6.8)	132 (7.9)	143 (7.8)	145 (7.5)

^a^Survivor function (probability of adherence) is calculated over full data and evaluated at indicated times; it is not calculated from aggregates shown in this table

Among both mothers and infants, a higher proportion of nonadherence events were due to stopping treatment not according to national guidelines (range: ART analysis: 73.9–88.4%, NVP analysis: 58.3–100%) rather than missing at least 5% of daily doses (range: ART analysis: 15.2–26.2%, NVP analysis: 0–41.7%) (Table 2).

### Risk factors of nonadherence to ART among mothers

Maternal age influenced ART adherence from 6 weeks to 18 months postpartum with adolescents and young women (aged 16–24 years) having a higher hazard of nonadherence events during that period than women over 34 (age 16–24, adjusted Hazard Ratio (aHR): 1.9, 95% CI 1.4–2.5).

Failing to disclose one’s HIV positive status to anyone at baseline was a risk factor for nonadherence (aHR: 1.7, 95% CI: 1.3–2.1). Not knowing one’s CD4 result also increased risk of nonadherence among mothers. Compared to mothers with a known CD4 result, those who had a CD4 test done but results were not received (aHR: 1.3, 95% CI: 1.1–1.6) or it was not known if CD4 test was done (aHR: 2.1, 95% CI: 1.4–3.2) were at higher risk for nonadherence.

Mothers who initiated ART after delivery were at higher risk for nonadherence (aHR: 1.6, 95% CI: 1.3–2.0) than those who initiated ART before delivery. Mothers who delivered their infants outside of health facilities had almost double the hazard of nonadherence than those who delivered at a facility (aHR: 1.9, 95% CI: 1.2–3.0), however only 43 mothers delivered outside of the facility.

Parity and whether the pregnancy was planned were included in the model but were not significantly associated with nonadherence.

### Risk factors of nonadherence to Nevirapine among infants

Provincial variation in NVP nonadherence in the 6 week to 18 month postpartum period was seen in the infant population with those in Northern Cape, North West, Western Cape, and Eastern Cape experiencing the highest hazard of nonadherence compared to those in Free State (Northern Cape, aHR: 5.3, 95% CI: 1.9–14.8; North West, aHR: 4.8, 95% CI: 1.8–12.6; Western Cape, aHR: 4.1, 95% CI: 1.4–11.7; Eastern Cape: aHR: 3.2, 95% CI: 1.1–9.3) (Table 3).

**Table 3:** Unadjusted and adjusted risk factors for nonadherence to ART among a cohort of mothers and NVP among a cohort of infants in South Africa followed in 2012–2014 identified with cox proportional hazards model (unweighted)

Mothers *N* = 1322^a^	Nonadherence Cox proportional hazard model					
Characteristic	Unadjusted	(95% CI)	*p* value	Adjusted	(95% CI)	*p* value
	hazard ratio			hazard ratio		
Maternal age						
35+ years	1			1		
25–34 years	1.21	0.96–1.54	0.111	1.21	0.95–1.54	0.123
16–24 years	1.93	1.46–2.53	0.000	1.87	1.42–2.47	0.000
Delivery care location						
Hospital/clinic	1			1		
Home/other	1.61	1.03–2.53	0.036	1.87	1.19–2.94	0.007
CD4 result received						
CD4 test done and result received	1			1		
CD4 test not done	1.43	0.88–2.34	0.150	1.34	0.82–2.20	0.242
CD4 test done, result not received	1.33	1.10–1.62	0.004	1.28	1.06–1.57	0.012
Do not know if had CD4 test	2.56	1.73–3.80	0.000	2.15	1.44–3.20	0.000
HIV status disclosed to family and/or friends						
Yes	1			1		
No	1.69	1.33–2.14	0.000	1.67	1.31–2.12	0.000
Planned pregnancy						
Yes	1			1		
No	1.25	1.04–1.51	0.017	1.18	0.98–1.42	0.082
No response	2.14	0.30–15.27	0.448	2.54	0.35–18.28	0.356
Initiated ART after delivery						
No	1			1		
Yes	1.66	1.36–2.03	0.000	1.61	1.31–1.96	0.000
Don’t know/Chose not to answer	1.15	0.83–1.59	0.404	1.14	0.82–1.58	0.425
Infants *N* = 1231^b^	Nonadherence Cox proportional hazard model					
Characteristic	Unadjusted	95% CI	*p*-value	Adjusted	95% CI	*p*-value
	hazard ratio			hazard ratio		
Province						
Free State	1			1		
Limpopo	1.30	0.35–4.86	0.693	1.31	0.35–4.89	0.689
Eastern Cape	2.97	1.01–8.68	0.047	2.97	1.01–8.73	0.048
Northern Cape	5.27	1.92–14.44	0.001	5.16	1.86–14.28	0.002
Gauteng	1.23	0.40–3.75	0.719	1.25	0.41–3.82	0.701
KwaZulu Natal	1.97	0.68–5.66	0.210	2.02	0.70–5.85	0.192
Mpumalanga	2.58	0.89–7.42	0.079	2.48	0.86–7.17	0.093
Northwest	4.86	1.85–12.79	0.001	4.81	1.82–12.72	0.002
Western Cape	4.28	1.48–12.32	0.007	3.95	1.37–11.41	0.011
Maternal parity						
One child	1			1		
2–3 children	1.26	0.74–2.13	0.394	1.07	0.62–1.82	0.817
4+ children	1.68	0.90–3.16	0.106	1.45	0.76–2.75	0.259
Mother ever heard of PMTCT						
Yes	1			1		
No	1.76	1.01–3.06	0.044	1.70	0.96–3.00	0.068
Mother knows partner’s HIV status						
Yes	1			1		
No	1.48	0.98–2.24	0.061	1.46	0.97–2.21	0.072

^a^Mothers were included in survival analysis if they were on ART at 6 weeks and had information about ART adherence at any follow-up visit

^b^Infants were included in survival analysis if they were on NVP at birth and had information about NVP adherence at any follow-up visit

Infants whose mothers had not heard of PMTCT prior to the six week interview had higher hazard of nonadherence within the 6 week to 18 month postpartum period than those whose mothers had heard of PMTCT (aHR: 1.6, 95% CI: 0.9–32.8; *p* value < 0.10), although this was not significant at 5%. Not knowing the mother’s partner’s HIV status showed increased hazard for nonadherence (aHR: 1.4, 95% CI: 10.9–2.1; *p* value < 0.10) but this was not significantly at 5%. Maternal parity was included in the model but was not significantly associated with adherence.

## Discussion

### Adherence to antiretroviral treatment among mothers and infants until 18 months postpartum

This study observed suboptimal ART adherence until 18 months postpartum among this cohort of HIV-positive mothers and infants, with below 65% of mothers and below 75% of infants cumulatively maintaining adherence over 95% from 6 weeks to 18 months postpartum. This is gravely concerning, given the global policy shift to lifelong ART amongst pregnant and lactating women, and the need for extended infant prophylaxis amongst mothers who are not virally suppressed. We found ART adherence to be higher at earlier time points, with both mothers and infants over 85% adherent by 14 weeks over or about 80% by 6 months. A similar analysis of infant ART adherence in Zambia showed a lower adherence until 6 months of about 70% (versus our result of 93.0%), and until 18 months of just above 0% (versus our result of 73.4%) [36]. A study from Malawi showed lower maternal ART adherence than our study, with 75% adherence by 3 months, about 50% adherence by 6 months, and under 40% adherence by 18 months [37]. While the study in Malawi used a pharmacy-based adherence measure, our study utilized self-reported adherence which tends to overestimate adherence due to recall bias and social desirability. This may account for the differences in adherence seen across these two studies. Results from a systematic review of adherence among women on ART in low- middle- and high-income countries reported adherence of 53% (95% CI: 33–73) during the postnatal period, although the timeframe was not specifically defined [4]. While our results from this cohort of mothers and infants indicate higher ART adherence than that seen in similar studies from the region, possibly because of the Hawthorne effect [38] as a result of three-monthly follow-up by nurses, adherence reported herein remains suboptimal to eliminate MTCT and highlights room for improvement on the implementation of PMTCT guidelines [5, 24].

Infant adherence was higher than mother adherence by about 10% across the entire 6 weeks to 18 months postpartum period. This may suggest that efforts made by the mother to ensure adherence within the mother-infant pair are prioritized toward the infant’s HIV prophylaxis adherence over her own HIV treatment.

### Risk factors of nonadherence to ART among mothers

We identified risk factors for nonadherence to inform further improvements to PMTCT programming. Adolescents and young women in this cohort (age 16–24 years) had a higher hazard of nonadherence than women older than 25, consistent with similar studies about PMTCT and maternal and child health seeking [39–41]. A study from Uganda indicates that this is potentially due to lack of knowledge and experience with childbirth and health, as well as age-related discrimination from the heath system [42]. In South Africa, adolescent girls and young women have particularly high risk for acquiring HIV infection and low uptake of HIV services [43]. Targeted HIV interventions to increase HIV prevention and service coverage among this high risk group were initiated in South Africa in 2015.

Initiating ART after delivery was a risk factor for nonadherence among mothers, indicating that initiation on ART earlier in the continuum of care may be an important protective measure to improve adherence. The current national guidelines entitling all HIV positive pregnant and breastfeeding women to lifelong ART partially address this concern [5]. Additionally, messaging that encourages timely attendance to antenatal care visits among pregnant women in South Africa may increase early initiation on ART [44].

Knowledge of one’s CD4 count protected mothers from nonadherence, indicating that compliance with CD4 testing and returning of test results to patients may have contributed to improved adherence to ART among mothers. At the time of these data, ART initiation was based on CD4 count< 350 under Option B guidelines, therefore for mothers who were not experiencing symptoms and therefore did not feel the pressing need for ART adherence, CD4 results may have offered a motivating factor [45, 46]. Counseling messages from health providers received with CD4 test results could have improved a mother’s understanding of the importance of ART adherence to PMTCT, thus reducing nonadherence [47]. This confusion around CD4 count is no longer an anticipated barrier at the present era of test and treat for life.

Disclosure of HIV status to family and friends was protective from nonadherence within this cohort, as seen in other studies from South Africa and other sub-Saharan African settings [48–50]. These studies postulated that the psychosocial support gained through disclosure, as well as the accountability to one’s treatment, encourage adherence. PMTCT programmatic messages should encourage mothers to disclose their HIV status to improve ART adherence. Furthering the case for support as a protective factor against nonadherence is the result that married and cohabitating mothers showed lower nonadherence than single, divorced, and widowed women.

Mothers who delivered their infants in health facilities had lower hazard of nonadherence than those who delivered outside of a formal health facility. We postulate that this is due to additional support provided when a delivery is in a health facility. Other studies have found that individuals exhibiting high health seeking behavior have been found to have higher adherence to ART [51]. Messages about the importance of ART adherence for PMTCT are provided whenever a mother interacts with the health system along the continuum of care [12], thus those delivering in a facility receive a higher frequency of these messages.

### Adjusted risk factors of nonadherence to Nevirapine among infants

Provincial variation in nonadherence may be indicative of inconsistencies in effectiveness of PMTCT, potentially explained by a complex network of differences in relative strength of the provincial health systems, messaging surrounding the importance of PMTCT, the general status of health, and geography. As a highly dynamic and migratory population, variation in health seeking behavior is expected, as noted in other studies [17, 43, 52].

### Limitations

By nature of the population followed until 18 months postpartum by the national PMTCT Evaluation study, the present study did not include adherence for HIV-exposed infants who had a positive PCR result at 6 weeks or their mothers. As a cohort analysis observing a subgroup from this follow-up population, the results were not adjusted for the study design, non-response and not weighted for live-births, thus limiting the representativeness of these results.

The Andersen-Gill extended cox proportional hazards model selected for this analysis requires a strong statistical assumption of independence across the increments in which nonadherence events can occur [35]. This assumes that a nonadherence event is not influenced by a prior nonadherence event within a study participant. Thus, event dependence is not included in the model. Without strong evidence to support dependence across nonadhernece events, we chose this model.

ART adherence was evaluated on self-reported responses, therefore issues of false reporting and recall likely biased the results, however we cannot determine whether these are under- or over-estimates. To minimize such information bias we checked road-to-health cards to verify maternal self-report of HIV status and ART uptake. The consistency between maternal self-report of these measures and the record from the road-to-health card increased our confidence in other self-reported measures.

A high proportion of mothers and infants included in the follow-up study missed interviews during the 18 month follow-up period. Due to the lack of information about these participants at intervals where the interview was missed, participants were not included in the analysis for any missed interval, likely introducing bias into the results. There was no date of failure captured for mothers who stopped taking ART during an interval, thus the midpoint was used which could have under- or over-estimated the time contributed.

A higher probability of adherence during later analysis time may been driven by loss to follow-up of non-adherent women and may not reflect an actual improvement in adherence, as women with poor adherence are at higher risk of loss to follow-up than those with good adherence [37, 53]. This mechanism of “informative censoring” biases analysis of adherence patterns over time.

The population studied herein is highly dynamic [17, 54, 55], exemplified by the fact that individuals could move in and out of the included study population at each follow-up interval. Such mobility likely contributes to the low adherence seen here, both by providing a barrier to adherence among mothers and their infants and by posing a challenge to measurement within our follow-up interviews. The high proportion of missed interviews underscores the importance of strengthening counseling to ensure that individuals understand the importance of ART adherence to viral suppression, reducing risk of MTCT, and reducing risk of drug resistance. Mothers were continually counseled on the importance of adherence to health outcomes throughout the course of the study, especially when non-adherence events were identified.

## Conclusion

Our findings from this cohort of mothers and infants in South Africa suggest that suboptimal adherence to ART for PMTCT persists, which likely contributes to remaining mother-to-child HIV transmission events. Results from this cohort of mothers and infants in South Africa suggest that young mothers are an important target for the PMTCT national program with messages stressing the importance of adherence; all pregnant women and mothers need support to disclose their HIV status to loved ones, to deliver in a facility, and to know their CD4 count to improve ART adherence. Additionally, a mother’s knowledge of PMTCT and knowledge of her partner’s HIV status could improve infant adherence to Nevirapine.

## Data Availability

Not applicable.

## Abbreviations

aHR: adjusted Hazard Ratio
ANC: Antenatal care
ART: Antiretroviral therapy
EMTCT: Elimination of mother-to-child transmission of HIV
HIV: Human immunodeficiency virus
MTCT: Mother-to-child transmission of HIV
NVP: Nevirapine
PLHIV: People living with HIV
PMTCTm: Prevention of mother-to-child transmission of HIV
WHO: World Health Organisation
